# Application of Bonelike® as synthetic bone graft in orthopaedic and oral surgery in veterinary clinical cases

**DOI:** 10.1186/s40824-018-0150-x

**Published:** 2018-12-29

**Authors:** José Miguel Campos, Ana Catarina Sousa, Pedro Olivério Pinto, Jorge Ribeiro, Miguel Lacueva França, Ana Rita Caseiro, Mariana Vieira Branquinho, Sílvia Santos Pedrosa, Carla Mendonça, Ana Brandão, José Domingos Santos, Américo Afonso, Luís Miguel Atayde, Ana Lúcia Luís, Ana Colette Maurício

**Affiliations:** 1 Escola Universitária Vasco da Gama (EUVG), Hospital Veterinário Universitário de Coimbra, Coimbra, Portugal; 2 Departamento de Clínicas Veterinárias, Instituto de Ciências Biomédicas de Abel Salazar (ICBAS), Universidade do Porto (UP), Rua de Jorge Viterbo Ferreira, n° 228, 4050-313 Porto, Portugal; 3 Centro de Estudos de Ciência Animal (CECA), Instituto de Ciências, Tecnologias e Agroambiente da Universidade do Porto (ICETA), Porto, Portugal; 4 REQUIMTE/LAQV – U. Porto – Porto/Portugal, Departamento de Engenharia Metalúrgica e Materiais, Faculdade de Engenharia, Universidade do Porto, Rua Dr. Roberto Frias, s/n, 4200-465 Porto, Portugal; 5 Biosckin, Molecular and Cell Therapies S.A., Laboratório Criovida, TecMaia, Rua Engenheiro Frederico Ulrich 2650, 4470-605 Moreira da Maia, Portugal; 6 Faculdade de Medicina Dentária da Universidade do Porto (FMDUP), 4200-393 Porto, Portugal

**Keywords:** Biomaterial, Bonelike®, Synthetic bone graft, Bone regeneration, Case series, Veterinary, Canine, Feline, Orthopaedics, And odontology

## Abstract

Autologous bone remains the gold standard grafting substrate for bone fusions used for small gaps and critical defects. However, significant morbidity is associated with the harvesting of autologous bone grafts and, for that reason, alternative bone graft substitutes have been developed. In the present case series, a glass-reinforced hydroxyapatite synthetic bone substitute, with osteoinductive and osteoconductive proprieties, was applied. This synthetic bone substitute comprises the incorporation of P_2_O_5_-CaO glass-based system within a hydroxyapatite matrix, moulded into spherical pellets with 250-500 μm of diameter. A total of 14 veterinary clinical cases of appendicular bone defects and maxillary / mandibular bone defects are described. In all clinical cases, the synthetic bone substitute was used to fill bone defects, enhancing bone regeneration and complementing the recommended surgical techniques. Results demonstrated that it is an appropriate synthetic bone graft available to be used in veterinary patients. It functioned as a space filler in association with standard orthopaedic and odontological procedures of stabilization, promoting a faster bone fusion without any local or systemic adverse reactions. This procedure improves the animals’ quality of life, decreasing pain and post-operative recovery period, as well as increasing bone stability improving positive clinical outcomes.

## Introduction

With increasing average life expectancy [1] the number of degenerative diseases, osteogenic disorders and bone fractures has scaled in recent decades [2]. Bone defects due to trauma, pathological and physiological bone resorption represent a major challenge and are a global health problem [3], in both human and domestic animal individuals. The traditional methods for repairing bone defects (such as autografts, allografts and xenografts) bare noteworthy disadvantages, thus limiting their clinical application [4, 5]. Some of these bone grafts can be resorbed in a time controlled way in order to allow the correct process of natural re-construction of the involved bone tissue to occur and restore skeletal integrity [3]. Large bone defect repair is a difficult problem to be solved urgently in the orthopaedic field and the application of bone repair materials appears as a viable alternative [6]. Recent development of calcium phosphate ceramics and other related biomaterials for bone graft provide improved control of the process of resorption and the ability for new bone to form. Glass-based ceramics, such as bioactive glasses, and calcium phosphates [calcium hydroxyapatite; tricalcium phosphate (TCP) and biphasic calcium phosphate] are popular materials for mineralised tissue regeneration [7]. Santos et al. demonstrated that hydroxyapatite bioactivity is improved through the combination with a P_2_O_5_-CaO-Na_2_O glass system (resulting biomaterial patented as Bonelike®) [8]. Bonelike® is composed by a modified hydroxyapatite (HA) matrix, with α- and β-tricalcium phosphate secondary phases, that counterpart HA’s brittle and essentially non-degradable character, improving the bioresorption of the modified system [9]. The structure of Bonelike® presents a computer controlled 3D architecture, and a complex composition that mimics the mineral composition of natural bone [10, 11]. Further, Bonelike® bares enhanced bioactivity by reproducing the inorganic phase of HA in bone which contains several ionic substitutions which modulate its biological behavior. Additionally, the mechanical properties of the temporary graft are improved by the CaO-P_2_O_5_ based glasses [12, 13].

The main indications for Bonelike® applications include non-unions, delayed unions, *mal* unions, bone defects, bone cysts, tumours and arthrodesis [14], and its clinical application has been reported in craniofacial, oral/maxillofacial, orthopedic and dental surgery [15]. The clinical applications in maxillary bone defects indicated good bonding between newly formed bone and the Bonelike® granules [16].

In Veterinary Medicine, fresh cancellous bone graft remains the gold standard in veterinary orthopaedics for enhancing defect healing. Fortunately, the use of synthetic bone grafts has been increasing in the last two decades, and the application of β-TCP derivates, like Bonelike®, has already stepped out of preliminary pre-clinical assays to veterinary applications [17]. Due to the costs involved in the use of biomaterials, its use is still not as frequent as in Human Medicine. However, the evolution of social consciousness and attention to pets is reflecting in increased disposition of owners to invest in the treatment of their animals.

Several case reports detail on the application of bone substitutes in veterinary patients. Successful resolution of *pes varus* in two miniature Daschshunds has been reported, using a wedge of synthetic β-TCP to fill the gaps created by *tibial* corrective open osteotomies. According to Izamisawa et al [18], 2 months after the surgery the implanted edges were integrated with native bone. The bone plates and screws were removed after 4 months and, by then, the TCP wedges were completely resorbed, and the osteotomy bone remodelled. The use of TCP avoided the need of a second surgery to harvest autologous cancellous bone graft. The postoperative angles were corrected and maintained during the follow-up period. Morphologically, the body of the tibia in the affected hind limb nearly equalled the one found in healthy limbs [19]. In a similar approach, granules of β-TCP were mixed with fresh blood and used as void filler in subcritical-sized bone defects in long bones in 13 animals. All but one case achieved complete bone union, and radiographic bone ingrowth ranged from 75 to 100%. The study reported excellent clinical results confirming the biocompatibility and usefulness of β-TCP as a synthetic bone graft for moderate to large subcritical bone defects with initially expected good biological conditions such as blood supply and cellular activity [20]. Another case report refers to a distal radius atrophic non-union in a one-year old male Yorkshire Terrier, using a 3D-printed β-TCP scaffold with bone morphogenic proteins (rhBMP-2) (truScient®) to create a scaffold with the same shape as the defect. After the removal of the bone plate, the scaffold was no longer visible and complete corticalization of the regenerated bone area was observed on computerized tomography (CT) scan evaluation. The scaffold was deemed an excellent bone substitute due to good osteoinductive properties of rhBMP-2, complemented with good osteoconductive potential provided by the open-interconnected pores from the β-TCP scaffold [20].

Herein we report a case series of 14 appendicular bone defects and maxillary / mandibular bone defects in which the surgical approach included the use of Bonelike® as a bone substitute, aiming at the optimization of the selected intervention outcomes.

## Materials and methods

### Case selection and general procedures

The case series includes 14 orthopaedic clinical cases in small animals, ten canine and four feline patients, presenting moderate to severe non-critical bone defects.

Feline patients ranged from 4 to 16 years of age with an average age of 8 years, weighing 3 to 6 kg (average 4.25 kg), 2 females and 2 males. Canine patients were aged 1 to 14 years (average age of 7.7 years), weighing 2 to 30 kg (average 18.5 kg), 1 female and 9 males. Two of the canine cases were appendicular bone fractures and the remaining were maxillary and mandibular bone fractures. Legal tutors of all animals provided informed consent on the application of Bonelike® as part of the instituted therapy. Patients were selected strictly on the basis of their clinical needs and according to the following criteria, which included: patients of any age, gender or weight, patients without any systemic disease/ infection, and finally, all clinical situations in which the time of bone healing was expected to supplant the standard time. The exclusion criteria included the presence of systemic disease, infected cavities, acute infection at the local of bone defect, bone inflammatory diseases (particularly osteomyelitis), malignant tumours, severe renal dysfunctions, animals with increased anaesthesia risk and animals with non-controlled bone metabolism. Individual data of each clinical case is summarized in Table 1: species, breed, sex, age, weight, diagnosis (motive for the surgery) and surgical procedure performed. Due to the disparity of animals’ size and affected bones, defects were graded/ classified empirically from small to large according to width and length of the defects [rather than being attributed a metric classification].

**Table 1:** Individual data of the animals included in the case series

Case n° #	Breed	Sex – Age – Body weight	Problem	Bone defect grading	Surgical procedure (reduction system)	Consolidation time of the lesion (weeks)	Functional Recovery	Skeletal system	Gender
1	**Spitz**	Male - 1y - 5 kg	Non-union defect of radius and ulna after two previous surgical failure	Large	Corrective osteotomy of radius with osteosynthesis plate in radius and another plate in ulna	12	Good	Appendicular bone defects	Canine
2	**Labrador retriever**	Male - 3y – 30 kg	Traumatic tibia fracture with a non-union defect after one previous surgical failure	Large	Tibia fracture repair with osteosynthesis plate	12	Good		
3	**Mixed breed**	Female - 8y - 25 kg	Traumatic mandibular fracture with a delay healing after one interdental wiring failure	Small	Mandibular reconstruction with osteosynthesis plate	8	Excellent	Maxillary and mandibular bone defects	
4	**Collie**	Male - 10y - 15 kg	Traumatic rostral mandibular fracture with loss of canine tooth	Medium	Mandibular reconstruction with external skeletal fixation	5	Excellent		
5	**Yorkshire Terrier**	Male - 4y - 2 kg	Periodontic-endodontic disease	Small	Reconstruction of alveolar cleft after a maxillary tooth extraction	4	Excellent		
6	**Mixed breed**	Male - 14y - 9 kg	Periodontic-endodontic disease	Small	Unilateral reconstruction of alveolar cleft after exodontic procedure	6	Excellent		
7	**Mixed breed**	Male – 15y -7 kg	Periodontal disease (oronasal communication)	Medium	Reconstruction of alveolar cleft after total premolar and molar tooth removal	12	Excellent		
8	**Pit Bull**	Male - 11y - 28 kg	Canine tooth fracture	Large	Extraction of one canine tooth	12	Excellent		
9	**Labrador retriever**	Male - 3y - 28 kg	Canine tooth fracture	Small	Extraction of one canine tooth	8	Good		
10	**Labrador retriever**	Male – 8y – 34 kg	Molar extraction	Small	Extraction of one molar tooth	8	Good		
11	*European Shortair*	Male - 7y - 4 kg	Periodontic-endodontic disease	Medium	Reconstruction of alveolar cleft after total premolar and molar tooth removal	12	Excellent		Feline
12	*European Shortair*	Female - 5y - 6 kg	Periodontitis (root reabsorption)	Small	Reconstruction of alveolar cleft after a maxillary tooth extraction	4	Excellent		
13	*European Shortair*	Male - 16y - 4 kg	Traumatic mandibular fracture	Small	Fracture repair with external fixation	12	Good		
14	*European Shortair*	Female - 4y - 3 kg	Traumatic mandibular fracture	Small	Fracture repair with external fixation	12	Good		

Bold data are canine patients. Italic data are feline patients

All surgical procedures were performed at the Veterinary Hospital of the University of Coimbra (HVUC) and the Veterinary Hospital of ICBAS from the University of Porto (UP-Vet). The animals were maintained at the referred hospitals and were fed twice a day with appropriate diet.

Before surgery, preventive food and water withdrawal was instituted for 8 and 2 h respectively. Preoperatively, patients were administered non-steroid anti-inflammatory drugs and opioids. Propofol was administered intravenously as general anaesthetic induction (dose adjusted to desired effect), and adequate anaesthesia depth maintained using sevoflurane. The surgical sites were shaved as needed and then scrubbed with disinfection solution. All the surgical approaches were undertaken in accordance to Halsted’s surgical principles.

**Fig. 1:**
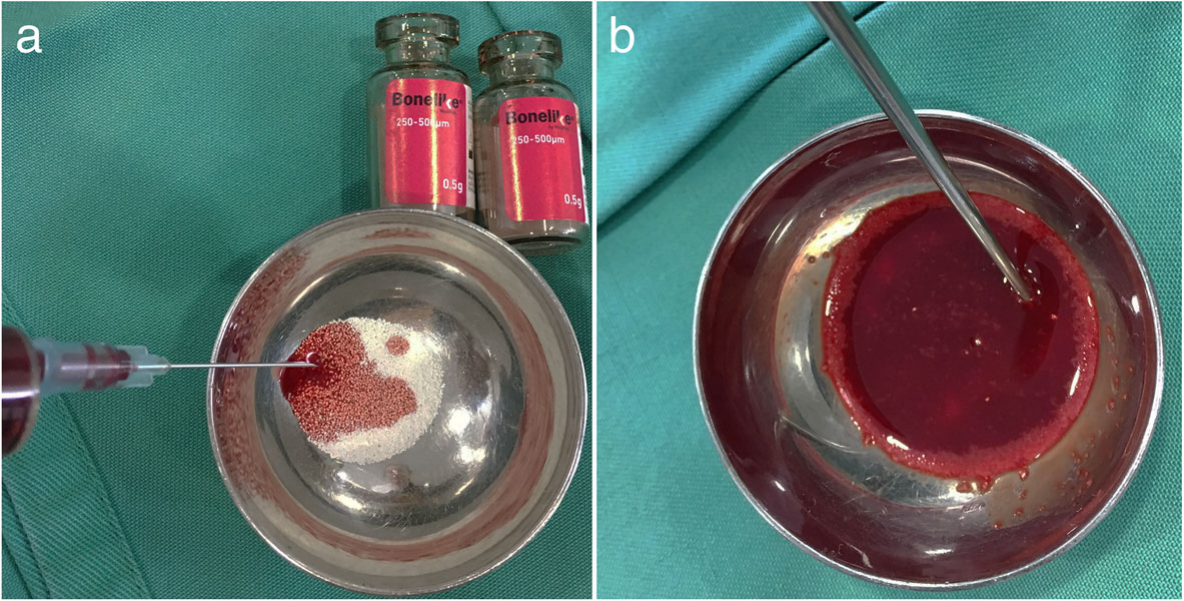
Preparation of Bonelike® mixture for intraoperative application, through mixture with autologous blood (**a**); mixed clot prepared for application (**b**)

In all small animal clinical cases included, Bonelike® was used to enhance bone healing, to fill critical defects and complement the recommended surgical procedures. In all cases, spherical granules of Bonelike® with a diameter between 250 and 500 μm were mixed with fresh autologous blood (Fig. 1). A single-ended curette helped to create the mixture and administer it to fill the bone defects.

After the required surgical intervention, tissue incisions were sutured in layers with absorbable Monosyn®. Postoperatively, opioids were administered to reduce pain for 2 days, anti-inflammatory drugs for 6 days, and beta-lactam antibiotics were administered for 8 days.

The follow-up consults included general physical examination, control X-rays and client-owned feedback in order to monitor the process. Radiographic monitoring was performed periodically and compared to preoperative records (kVp and mA settings individually adjusted to each case; files saved as DICOM). To evaluate functional recovery, a simple scale with three degrees was admitted: excellent, good and bad, indicating total, partial and no recovery of function, respectively.

### Clinical cases description

In clinical case #1, one-year-old toy breed dog (Spitz), with 5 kg of bodyweight (b.w.) presented a fracture of the radius and ulna with a large loss of bone, after a low-level trauma event. The patient was treated with an initial external skeletal fixation (modified type 2b) and subsequent neutralization osteosynthesis plate fixation. However, both corrective techniques applied failed.

**Fig. 2:**
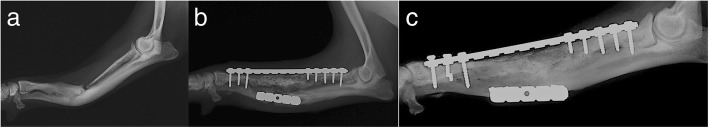
Case #1 - Latero-lateral radiographic images of the front limb, presenting a critical radio-cubital bone defect. Preoperative non-union after two events of implant failure (**a**); Postoperative control 4 months after surgical correction using Bonelike®(**b**); Postoperative control 8 months after surgical correction using Bonelike®(**c**)

On the third admission for surgery, the patient presented a severe atrophic non-union, classified as a critical large bone defect that was not expected to heal without additional intervention or bandage. Surgery was performed, and the synthetic bone graft Bonelike® was used, due to the limited availability of autologous cancellous bone for grafting. For the purpose, Bonelike® granules were mixed with autologous cancellous bone graft and deposited to fill the 45 mm-defect on the length of the radius, and a neutralizing bridge plate was applied. A complementary corrective ulnar osteotomy was performed with an additional neutralizing plate in order to improve bone alignment. Radiographic pre- and post-surgical control was performed and it is represented in Fig. 2. The functional recovery was good after 12 weeks, and radiographic control indicated good mineralization of the defect area and visible lines of ossification.

**Fig. 3:**
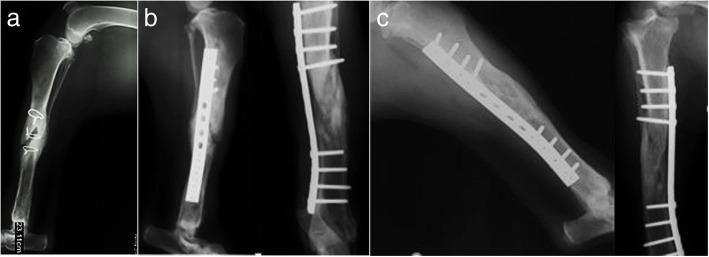
Case #2 – Latero-lateral and craniocaudal radiographic images of the hind limb, presenting a critical *tibial* bone defect. Preoperative non-union after internal fixation failure (**a**); Immediate postoperative control after surgical correction using Bonelike®(**b**); Postoperative control 4 months after surgical correction using Bonelike®(**c**)

In clinical case #2, a 3-year-old male Labrador Retriever, weighing approximately 30 kg b.w., presented a *tibial* fracture with large bone defect, after traumatic fracture. Surgical correction was attempted with internal fixation of the bone fragments with cerclage wiring, but bone fragment dislodgement resulted in failure of the surgical approach described. In a secondary surgical intervention, Bonelike® was applied on the *tibial* non-union, mixed with autologous cancellous bone, as well as crushed bone remnants and platelet rich plasma. Radiographic evaluation and clinical examination were performed in the follow-up periods (4, 8 and 12 months) to assess for the efficacy of the surgical treatments (Fig. 3). Clinical outcome for up to 12 months was graded as good and effective osteointegration was confirmed.

**Fig. 4:**
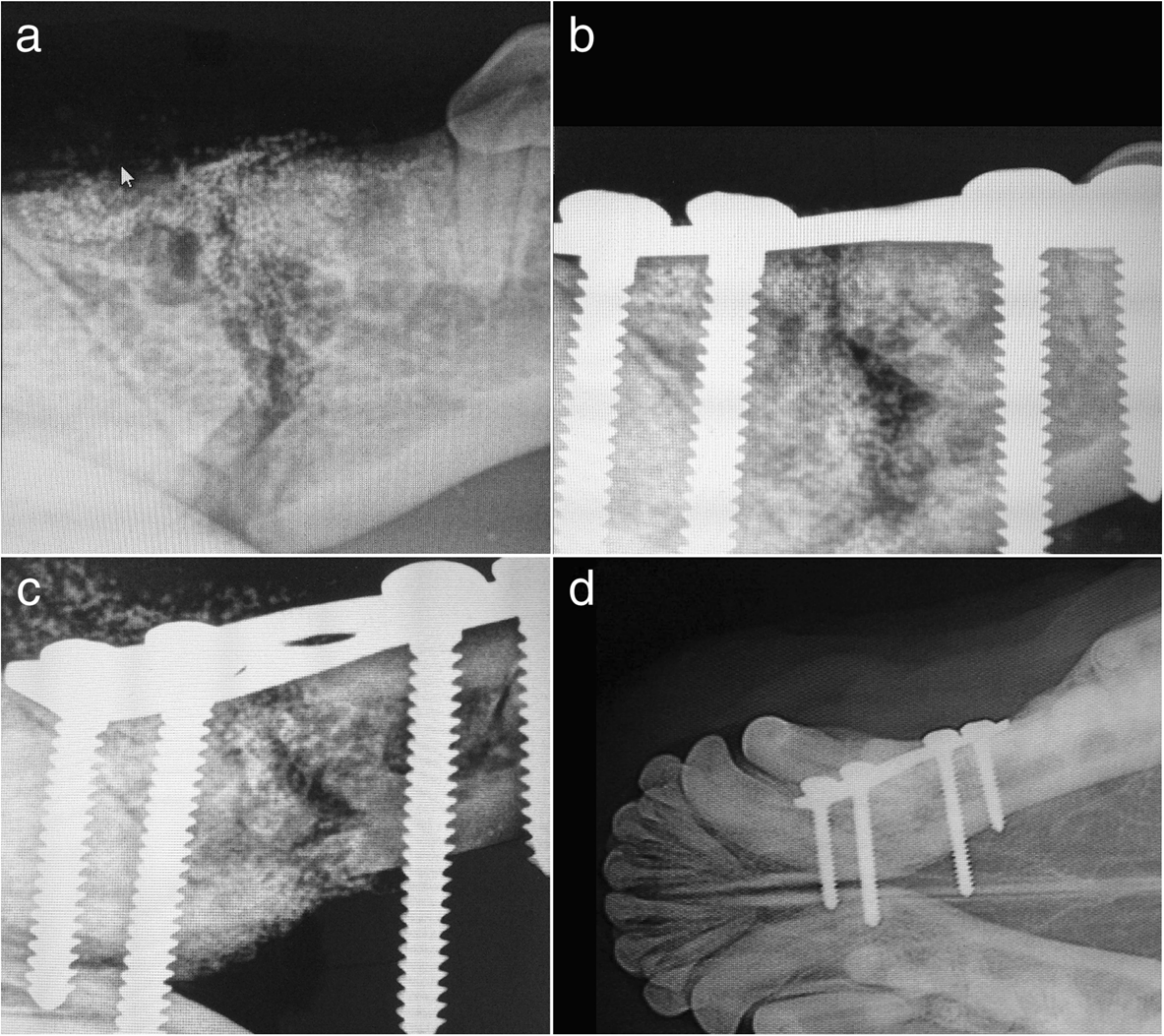
Case #3 - Radiographic study of the mandibula presenting an oblique fracture and absence of adjacent teeth (**a**); Immediate postoperative control after surgical reduction by plate fixation and Bonelike® filling of the mandibular alveolar bone (**b**, **c**); Postoperative control 8 weeks after surgical correction using Bonelike®(**d**)

In the clinical case #3, an 8-year-old female mixed-breed, with 25 kg b.w., presented with a complex traumatic oblique mandibular fracture, which was primarily treated with fixation of a mandibular fracture using interdental wiring. However, the fracture borders overlapped delaying the healing process. At a second intervention, the fracture was reduced with a neutralizing osteosynthesis plate and the maxillary alveolar bone defect filled with Bonelike® spherical granules. Orthopedic jaw fixation was performed using plate and screws to avoid damage to the root structures or disrupt the dental vascular supply. After 8 weeks, radiographic control fracture consolidation (Fig. 4) and a very good recovery of masticatory function was described by the legal tutors.

In clinical case #4, an 8-year-old male Collie, weighing 15 kg b.w., presented a fracture of the left side rostral jaw, resulting in canine tooth avulsion (affecting the tooth roots, nerves, blood vessels and salivary ducts) which created a medium size bone defect, between the incisor and premolar teeth. The mandibular fracture was repaired with an external fixation system, and Bonelike® spherical granules were used to fill the mandibular space resulting from structure avulsion.

**Fig. 5:**
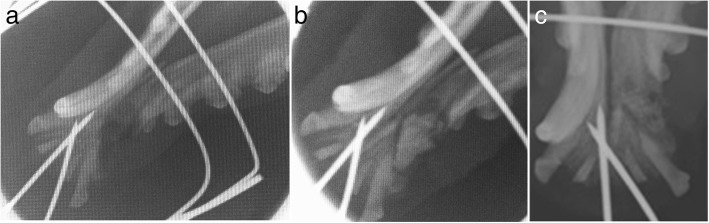
Case #4 - Radiographic study of the mandibula presenting a mandibular fracture and absence of adjacent teeth (**a**); Immediate postoperative control after external fixation and Bonelike® filling of the alveolar bone defect (**b**); Postoperative control 5 weeks after surgical correction using Bonelike® (**c**)

Follow-up consult after 5 weeks confirmed fracture consolidation and excellent ability to eat and drink was described by the legal tutors. The external fixation apparatus was removed uneventfully (Fig. 5).

**Fig. 6:**
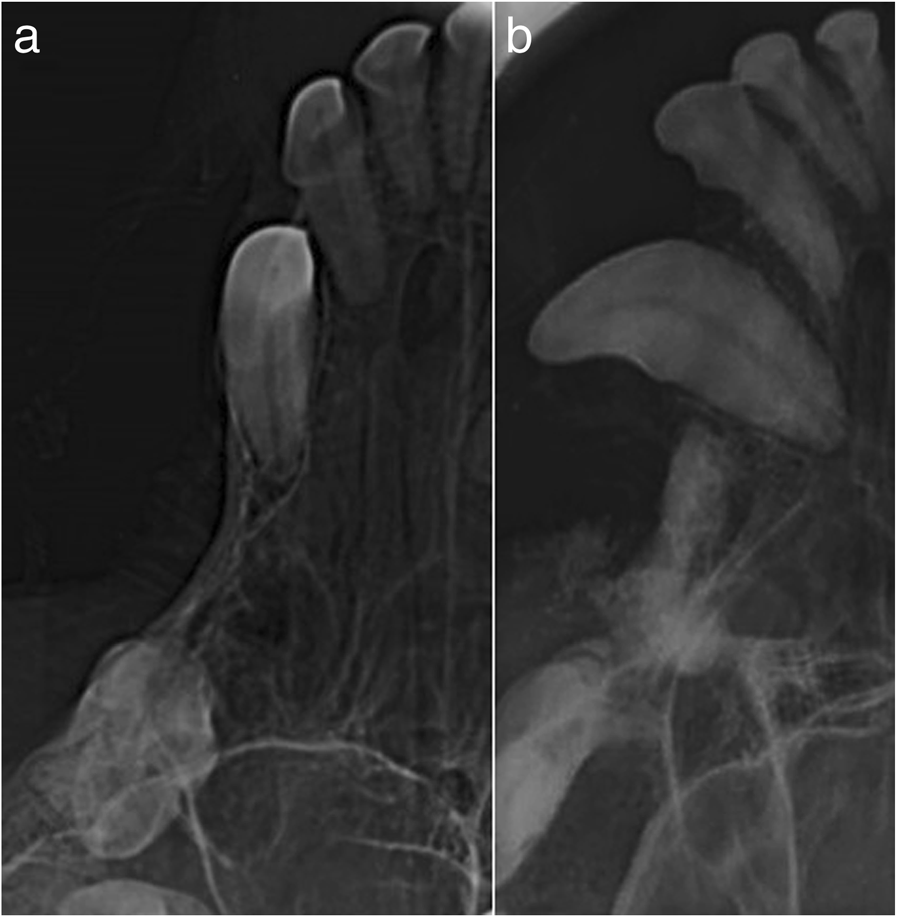
Case #5 - Radiographic study of the maxilla evidencing the alveolar defect resulting from exodontia of the 1st pre-molar tooth before (**a**) and after filling on the same with Bonelike® (**b**)

In clinical case #5, a 4-year-old small male Yorkshire Terrier was presented for consult due to progressive weight loss, weighing 2 kg b.w. at the time of the veterinary clinical evaluation. Upon consult and examination, additional clinical signs were identified, such as anorexia, dysphagia, apparently unspecific pain and an external root resorption of the maxillary pre-molar tooth, possibly progressing into crown destruction (suggested by a pink hue commonly associated to changes in mineral consistency). Radiographic examination highlighted a lucency area at the root tip of the maxillary tooth. An exodontic treatment was recommended, but the fragility of this maxilla was a concern. To address this fragility and to avoid spontaneous fracture, Bonelike® was applied after the extraction, to fill the defect and restore the maxillary bone integrity. After 4 weeks, the oral / gingival mucosa was healed and radiographic evaluation revealed complete and homogenous mineralization of the maxillary bone. No adverse reactions were detected and the patient regained appetite and normal food intake, demonstrating an excellent functional recovery (Fig. 6).

**Fig. 7:**
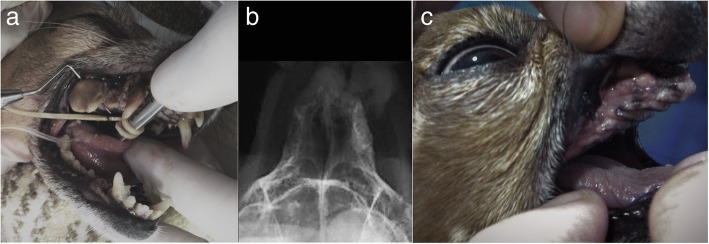
Case #6 – Visual examination of the oral cavity affected by severe periodontal disease (**a**); Postoperative control 6 weeks after exodontic procedure and filling of the left maxillary cavity using Bonelike® (right side of the image) (**b**); Visual examination of the oral cavity 6 weeks after exodontic procedure and filling of the left maxillary cavity using Bonelike®(**c**)

In clinical case #6, a 14-year-old mixed-breed presented with severe periodontic and endodontic disease of the maxillary molar teeth. Radiographic examination revealed severe periodontal bone loss, with profound periapical lucency, indicative of endodontic disease and periodontitis. Total extraction was carried out and small alveolar bone spaces were asymmetrically created on the maxillary bones. Application of spherical Bonelike® was performed exclusively on the left maxilla, where bone compromise was more impressive, to avoid future nasal fistula, and the right maxilla was kept unfiled. No adverse reactions were observed, and a smooth recovery was reported by the legal tutor. At 6 weeks post-intervention, the clinical examination revealed great improvement of buccal health and increased bodyweight. Moreover, a mineralized consolidation of the left grafted area where it was applied the Bonelike®, was confirmed through radiographic analysis (Fig. 7), while minor radiopacity was observed in the right side.

**Fig. 8:**
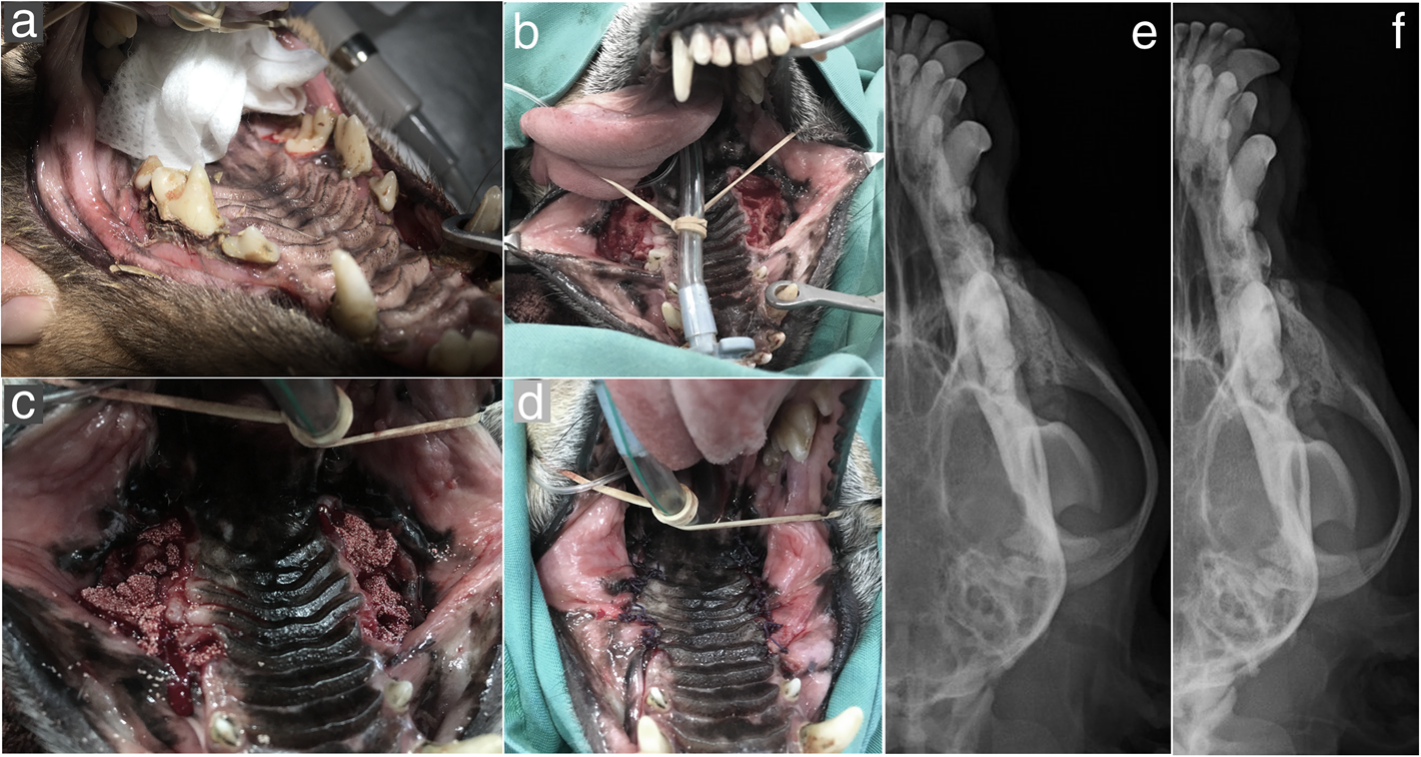
Case #7 - Visual examination of the oral cavity affected by severe periodontal disease (**a**); Surgical extraction of all teeth caudal to the 1st maxillary pre-molars, resulting in exposed maxillary alveolar cavities (**b**), that were filled with Bonelike®(**c**) and covered by a mucosal flap (**d**); Postoperative control 12 weeks after exodontic procedure and filling of the defect using Bonelike® (**e**: right maxilla, **f**: left maxilla)

In clinical case #7, a 15-year-old mixed-breed canine was admitted with severe periodontal disease, in a similar presentation to Case #6. The X-rays revealed a wide periodontal ligament space and a periapical lucency with teeth root resorption on both sides of the maxilla. The teeth that presented crown resorption had a pink hue due to the changes in the mineral composition of the crown. Extraction of all teeth caudal to the 1st maxillary premolar was advised. After the extraction, the alveolar bone defect was filled with Bonelike®, and a mucosal flap was sutured over the bone with no tension, to occlude a patent oronasal fistula (Fig. 8). Radiographic evaluation and clinical examination were performed 12 weeks after surgery to assess the efficacy of the surgical procedures. Adequate mineralization was observed, suggesting Bonelike® integration with the surrounding alveolar bone.

**Fig. 9:**
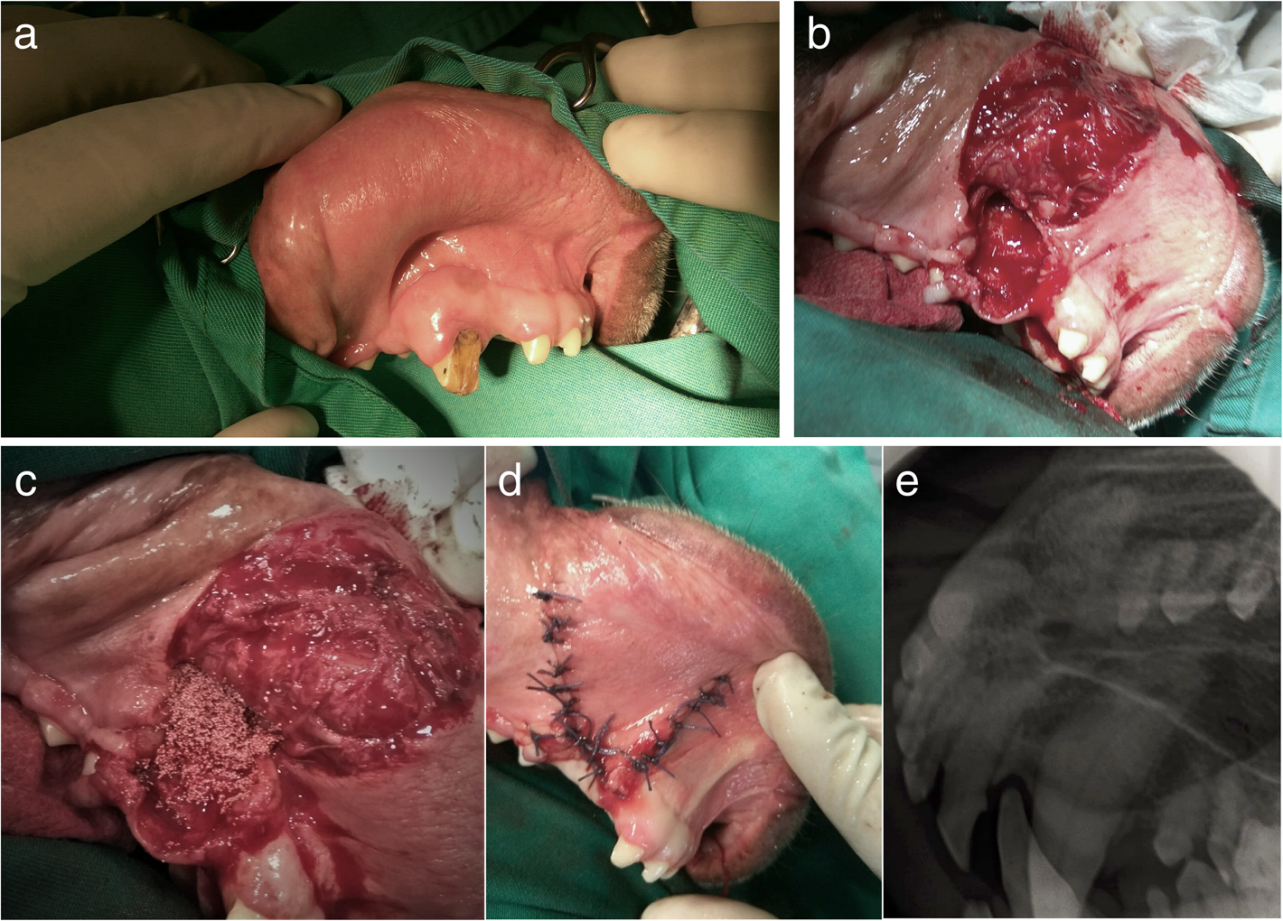
Case #8 - Visual examination of the oral cavity and identification of the fractured canine tooth (**a**); Exposed maxillary alveolar cavity after tooth removal (**b**), that was filed with Bonelike®(**c**) and covered by a mucosal flap (**d**); Postoperative control 12 weeks after exodontic procedure and filling of the defect using Bonelike®(**e**)

In clinical case #8, an 11-year-old male Pit Bull presented with a very common condition: a canine tooth crown fracture with no pulp exposure, and with periapical lucency surrounding the root upon radiographic assessment. Root canal therapy and crow reconstruction or extraction were the advised treatment options, as the maintenance of the damaged tooth would allow the bone destruction to progress. Pain was reported by the legal tutor as the main concern. Exodontia was selected as the treatment procedure, and once attained complete root exposition, the tooth, was removed. After the complete removal of the debris, the remaining defect was firmly packed with Bonelike® (mixed with autologous blood) to completely fill the cavity and cortical bone contour. Then a mucosal flap closure was performed, to prevent oronasal fistula formation. Care was taken to ensure sufficient mobility of the mucosal flap to cover the granules (Fig. 9). After 12 weeks the defect was completely mineralized, and the animal was eating well and without any concerns from the tutors.

**Fig. 10:**
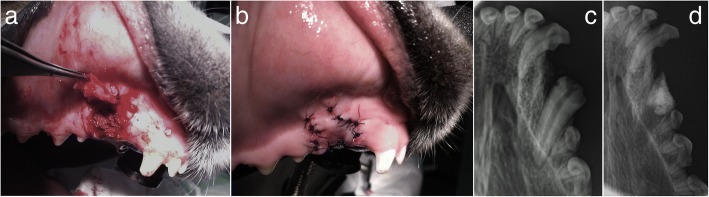
Case #9 - Visual examination of the oral cavity and identification of the fractured maxillary canine tooth (**a**); Exposed maxillary alveolar cavity after tooth removal that was covered by a mucosal flap (**b**); Preoperative control (**c**); Postoperative control after exodontic procedure, depicting the incomplete filling of the defect using Bonelike® (**d**)

In clinical case #9, a 3-year-old male Labrador Retriever presented with a similar defect under the maxillary mucosa of the canine tooth. Tooth extraction was performed and resulting bone cavity was filled with Bonelike®. Primary closure of the mucogingival flap was performed over the bone substitute filled defect. Immediate postoperative radiographic control indicated incomplete filling of the alveolar cavity with Bonelike® (Fig. 10). After 8 weeks the patient had an unremarkable examination of the mouth, showing complete recovery and bone regeneration of the maxillary defect.

In clinical case #10, a 8-year-old Labrador Retriever presented with a missing mandibular molar. Surgical exploratory surgery was undertaken, and the bone defect was filled with Bonelike® granules in order to promote the bone regeneration of the alveolar defect.

**Fig. 11:**
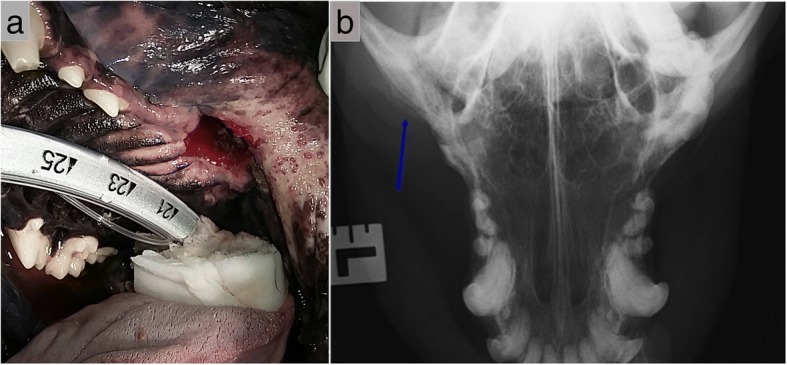
Case #10 - Visual examination of the oral cavity and the molar tooth absence (**a**); Postoperative control 8 weeks after surgical filling of the defect using Bonelike® (**b**)

Recovery was positive and, 8 weeks after surgical approach, good mineralization was evident. Radiographic imaging as well as animal’s behaviour and examination were unremarkable, confirming complete recovery and regeneration (Fig. 11).

**Fig. 12:**
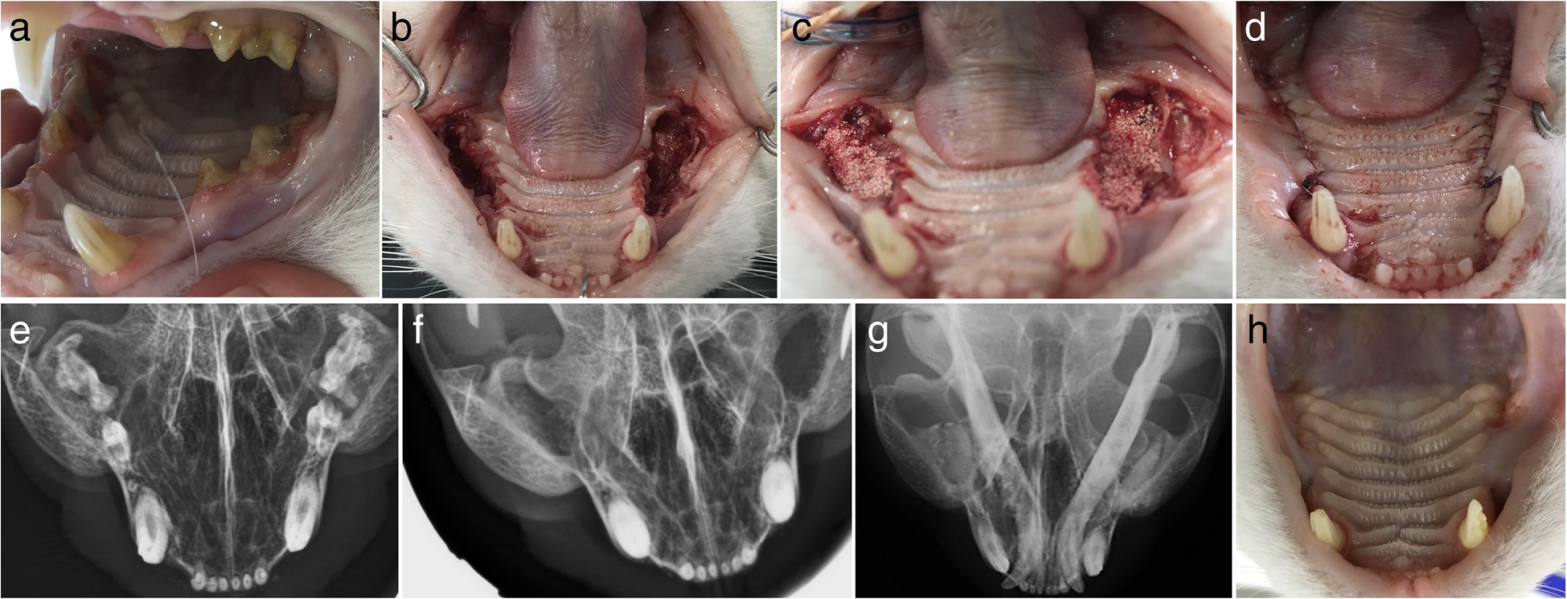
Case #11 - Visual examination of the oral cavity evidencing periodontal disease and stomatitis-gingivitis complex (**a**); Exposed maxillary alveolar cavities after molar and premolar extraction (**b**), that were filed with Bonelike®(**c**) and covered by a mucosal flap (**d**); Preoperative radiographic control; **f**: Intraoperative radiographic control after tooth extraction (**e**); Postoperative control 6 weeks after surgical filling of the defect using Bonelike®(**g**); Visual examination of the oral cavity 6 weeks after surgery (**h**)

In clinical case #11, a 7-year-old neutered male feline presented with periodontic-endodontic disease with associated stomatitis-gingivitis complex. After the extraction of molar and premolar teeth, spherical Bonelike® granules were applied in both maxillary alveolar defects. After 12 weeks, a good osteointegration was confirmed radiographically and no soft tissue nor systemic adverse reactions were detected (Fig. 12). Owners reported no pain, no swelling, no halitosis and overall a better quality of life.

**Fig. 13:**
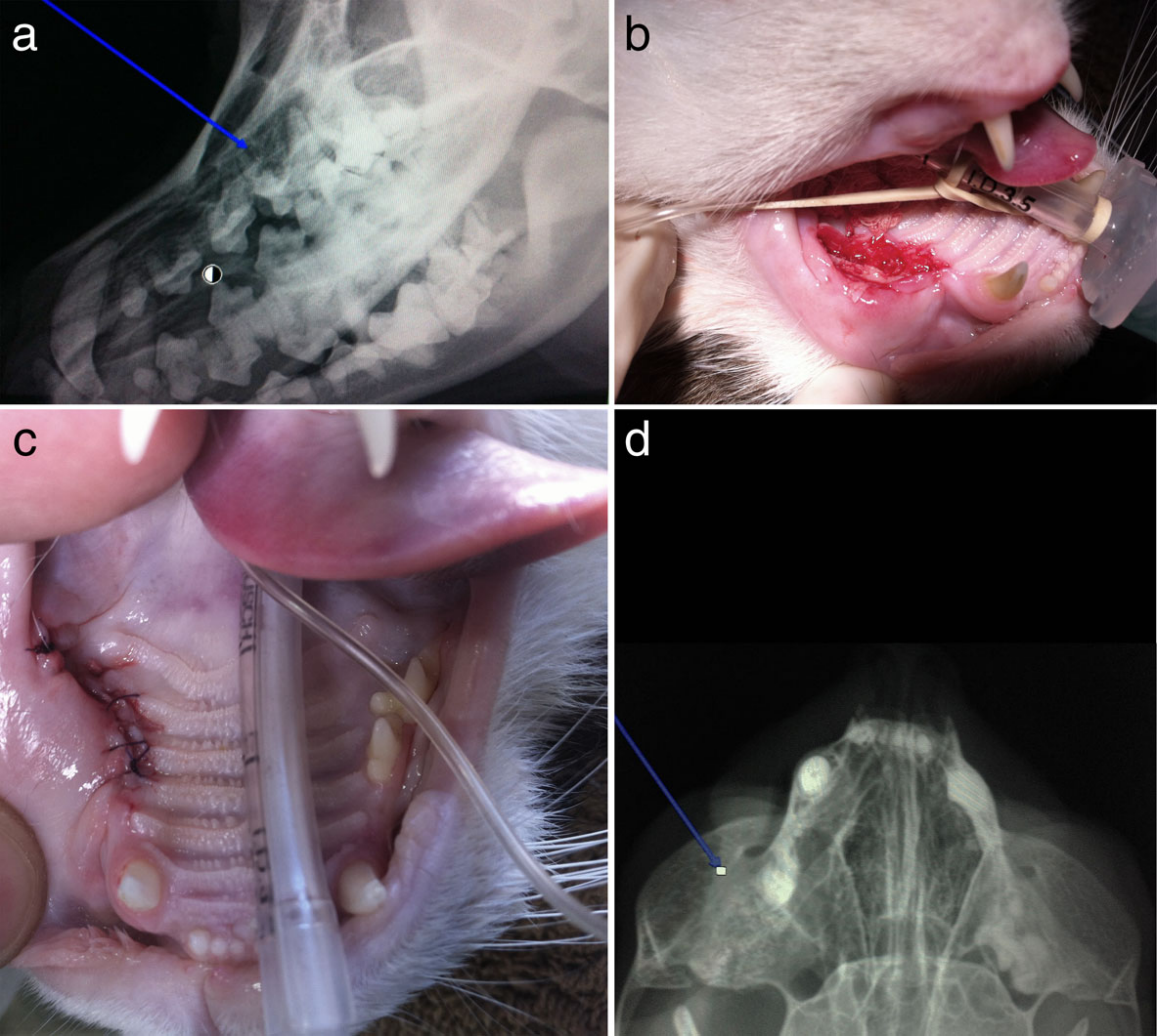
Case #12 -Radiographic study evidencing dental root resorption (blue arrow) (**a**); Small alveolar defect after premolar unilateral exodontia (**b**) that was filed with Bonelike® and covered by a mucosal flap (**c**); Postoperative control 4 weeks after surgical filling of the defect using Bonelike® (blue arrow) (**d**)

In clinical case #12, a 5-year-old female feline presented with periodontitis and several roots resorption. Clinically, the patient had developed progressive dysphagia. Conservative medical treatment with antibiotic and non-steroidal anti-inflammatory drugs was ineffective. Extensive maxillary tooth extraction was performed, and the alveolar cleft reconstructed with Bonelike®. An excellent functional recovery was observed and good behaviour recuperation was described (Fig. 13).

In clinical cases #13 and #14, feline patients presented with mandibular fractures. Treatment courses adopted were similar to those reported for the canine patients in Cases #3 and #4, through orthopaedic reduction and fixation of the fractures in combination to Bonelike® grafting of the bone gaps. Both patients recovered well with satisfactory radiographic controls, indicating adequate bone healing. Fixation apparatus were removed 12 weeks after surgery.

## Results

A series of 14 cases is herein reported, exploring the suitability of Bonelike® spherical granules of 250–500 μm diameter, in different types of bone defects in small animal clinical practice. In the detailed applications, Bonelike® was employed as bone substitute and to complement to traditional procedures, such as internal and external fixation of fractures, and iatrogenic bone voids.

Between the years of 2017 and 2018, the described veterinary clinical applications were performed in 10 canine and 4 feline patients, aged from 1 to 16 years (mean 7 years, median 8.5 years). These surgical procedures included Bonelike® grafting on defects after mandibular fractures with loss of bone and/or mandibular and maxillary tooth extractions (8 canine and 4 feline patients). Bonelike® applications described in the present manuscript, also included non-union fractures in long appendicular bones (2 canine patients).

Cases #1 and #2 presented with non-union defects of appendicular long bones, in canine patients, after previous surgical failures. Both resulted in good functional recovery after 12 weeks with the application of Bonelike® simultaneously to surgical reduction with osteosynthesis plates. Regarding maxillary and mandibular bone defects, 8 clinical cases were presented in canine and 4, in feline patients. Cases #3–4, and #13–14 were traumatic mandibular fractures in canine and feline patients, respectively. All were treated with external fixation except for case #3, in which an osteosynthesis plate was applied to the mandible. The remaining cases comprise Bonelike® application in alveolar defects, secondary to singular and multiple tooth extractions, to prevent oronasal fistula and imminent fractures. Through radiographic and physical examination, fracture consolidation was confirmed in all cases before 12 weeks.

Previous studies had proved Bonelike® biocompatibility in hard tissue applications [7], and no adverse systemic or local tissue reaction to Bonelike® were reported herein. Additionally, there was no record of post-operative infection, foreign body reaction or tear of the mucosa, regardless of the grafted defect size. Radiographic evaluation and clinical examination were performed in the follow-up periods in order to assess the efficacy of the surgical treatments associated to the bone substitute application. In all clinical cases, postoperative radiographic controls indicated high patterns of bone regeneration. All bone defects and extraction socket sites healed uneventfully for up to 12 weeks. At this point, the soft tissues enclosing the surgical sites had a healthy appearance and physiological consistency. In all odontological cases, grafts were stable and surrounded by healthy mucosa, contributing to a total functional recovery.

The radio-opacity of the Bonelike® in the immediate postoperative radiographic controls enabled its differentiation from the native bone tissue, and allowed us to accompany its integration with the surrounding tissues (that resulted in attenuation and diffusion of the grafted cavities, and progressive integration on native bone radiodensity). Nevertheless, Bonelike® presence was still evidenced throughout the follow-up period, with increased opacity. This observation is particularly denoted in Case #6, where Bonelike® was applied unilaterally and a comparable contra-lateral defect was left unfilled.

Most importantly, legal tutors of the presented patients reported significant improvement on the quality of life of their pets, recovering previously impaired functions (weight-bearing in the recovering limb or gripping and/or mastication capacity). The most relevant point reported by the legal tutors was the absence of pain. Using the empiric adapted functional recovery scale, clinical recovery was classified as excellent (80%) in most cases, whereas 20% were classified as good.

Unfortunately, tutor compliance with follow-up scheduling was suboptimal, leading to the inability to further accompany bone-Bonelike® interactions and long-term outcomes.

## Discussion

The physiologic reaction to a fracture is a spontaneous sequence of events briefly resumed as initial inflammation, followed by soft callus formation, hard callus formation, and ultimately bone remodeling [21]. When this natural process does not occur, as in the case of fracture non-union or large-scale traumatic bone injury, surgical intervention is warranted [22]. An insufficient blood supply and infection of the callus or the surrounding tissue or even systemic diseases can bare further negative effects on bone regeneration, resulting in a non-union [23]. The use of bone grafts is recommended in several surgical situations where the healing is difficult to achieve [24]. The consensual ‘gold standard’ graft remains the autograft, which does not induce immunological reactions and has the ability to provide osteoinductive growth factors, osteogenic cells, and acts as structural scaffold to new bone ingrowth [2, 22, 25]. However, this procedure is associated with prolonged anesthetic times, limited availability, donor site morbidity (pain, intra-operative blood loss and risk of stress fracture), risk of local infection and predisposition to failure [17]. Other issue is the limited amount of bone graft that must be collected from the animal and the viability of the cells after harvesting that limits the application to critical defects [25]. To address these problems, synthetic bone substitutes overcome some of the disadvantages listed for the autografts and can be used to fill critical voided spaces. These osteoinductive materials can be stored easily [22, 26, 27].The present case series report demonstrates Bonelike® spherical granules as an alternative to bone grafting techniques in the recovery of those patients, restoring the bio functionality of the affected area in small animals, in a similar manner to previously reported in maxillofacial surgery in human patients [28]. Bonelike® is an excellent ceramic scaffold to promote bone regeneration processes. It is made from an inorganic, nonmetallic material that can possess a crystalline structure [22]. Bonelike® contains a great proportion of HA, which is the main mineral component of the naturally occurring bone [29]. The complex composition of Bonelike® that combines HA, TCP and bioglass results in a tailored degradation rate, that accommodates new bone ingrowth while maintaining the grafted area integrity (the TCP phase has as faster degradation rate than the HA and bioglass phases) [7, 14].

In Case#1 a complimentary approach was adopted, due to the extension of the bone defect (and its critical nature), and an autologous cancellous bone graft was combined with Bonelike® spherical granules, demonstrating that compromise between bone and biomaterial grafting techniques may boost the clinical outcomes of complex cases, probably due to the addition of important growth factors and biomolecules present in the bone that promote the bone regeneration of the defects.

The remaining reported defects were of sub-critical size, and adequate bone healing was expected in defined periods of time (mandibular and maxillary bones 3-4 weeks; appendicular bones 8-12 weeks). Nevertheless, the application of the biomaterial filler aimed at the acceleration of the mineralization of the bone voids, thus decreased the risk for postoperative fractures associated with resulting bone fragility.

The maintenance of small granular biomaterial within a cavitary defect is often a challenge and mixing with an adjuvant matrix is often required. Mixing of Bonelike® with autologous peripheral blood and or platelet derived plasma achieved the desired effect in the detailed cases, avoiding biomaterial leakage. Additionally, the cellular populations enclosed within the formed clots are source for bioactive factors that activate intrinsic healing mechanisms at the defect site, and are proposed to further contribute to improved regeneration [30]. Other matrices would be interesting for the combined application, such as surgical fibrin glue, but associated costs may prevent widespread utilization, especially in the Veterinary Medicine field [31]. In the specific application of Bonelike® in oral surgery, mixture with the haemoderived matrix was reinforced by a mucosal flap, to further stabilize the Bonelike® spherical granules in the cavity and to protect the site from foreign bodies’ penetration (food, as an example). Currently, the goal was to fill the alveolar defect left by exodontic procedures. In similar applications in human patients, the target is the preparation of a congruent cavity in the alveolar process, thus promoting the integration between bone and future placed dental implant [32].

This surgical approach using a synthetic bone substitute, may be additionally combined with cell-based therapies, to potentiate the benefits of osteoregenerative biomaterials in orthopedic and oral surgery [33]. The combination of such cell-based therapies (dental pulp stem cells, for example) results in enhanced and accelerated regeneration processes [31].

Nevertheless, further prospective randomized clinical trials are essential to substantiate preliminary clinical case reports and provide quantitative and comparative data between the available and recently developed therapeutic strategies [9, 23].

## Conclusions

Autologous bone remains the gold standard grafting substrate available for bone fusions for small gaps and critical defects, but ceramic-based biomaterials, such as Bonelike® arise as a very adequate candidate biomaterial for application in orthopedic and oral surgery, in both humans and veterinary clinical scenarios. The present report details a series of 14 small animal clinical cases, including appendicular bone defects and maxillary / mandibular bone defects. In all clinical cases, Bonelike® application complement the recommended surgical techniques, and resulted in enhanced bone healing and total functional recovery. The positive defect healing and lack of tissue and systemic adverse effects observed allows us to also hypothesise Bonelike® as a structural scaffold for new-bone ingrowth. This strategy is available to be used in small animals as a space filler, and in association with standard orthopaedic and odontological procedures, thus promoting accelerated recoveries. This procedure improves the animals’ quality of life, decreasing pain and post-operative recovery period, as well as increasing bone stability improving positive clinical outcomes.

Nevertheless, clinical trials are required in Veterinary Medicine to adequately assess the outcome of the novel treatment options, establishing the most appropriate treatment protocols for each clinical presentation.

## Abbreviations

HA: Hydroxyapatite
TCP: Tricalcium phosphate
